# Evaluation of the adequacy of care for individuals with diabetes in Brazil: The intersection between gender, race, and socioeconomic profile

**DOI:** 10.1371/journal.pone.0349257

**Published:** 2026-08-07

**Authors:** Fábia C. G. M. Fernandes, Ricardo A. Bezerra, Isabelle R. Barbosa

**Affiliations:** Graduate Program in Public Health, Federal University of Rio Grande do Norte, Natal, Rio Grande do Norte, Brazil; Independent Global Health Consultant, INDIA

## Abstract

**Introduction:**

Diabetes is a major global public health problem. Inequity in access to healthcare services represents a significant challenge for healthcare management and particularly affects vulnerable populations, understanding the intersectionality of these social determinants of health is essential.

**Objective:**

To evaluate the adequacy of care provided to individuals with diabetes in Brazil, considering the intersection of gender, race, and socioeconomic profile.

**Methods:**

This study was based on data from the Brazilian National Health Survey (2019–2020). 6,967 individuals self-reported a diagnosis of diabetes and composed the study sample. The “adequacy of care” outcome variable was analyzed according to sociodemographic characteristics. The intersection of gender, race and socioeconomic profile was considered according to the intersectionality theory. Descriptive and multivariate analyses were conducted using Poisson regression (prevalence ratio [PR]) with robust variance and corresponding 95% confidence intervals (95%CI).

**Results:**

Black women with low income was the predominant profile of people with diabetes (22.0%; 95%CI: 20.5 to 23.3). Prevalent gaps in care delivery encompassed a lack of care for diabetic foot and failure to refer patients to a specialist doctor. The highest prevalence of low adequacy of care was observed in black men (79.6%; 95%CI: 75.7 to 83.1) and women (79.1%; 95%CI: 76.2 to 81.8) with low income. In the multivariate model, low adequacy of care was associated with black women and men with low income (PR = 1.09 [95%CI: 1.00 to 1.20] and 1.10 [95%CI: 1.00 to 1.21]), absence of diabetes-related complications (PR = 1.18 [95%CI: 1.11 to 1.25]), and no history of diabetes-related hospitalization (PR = 1.12 [95%CI: 1.03 to 1.22]).

**Conclusion:**

Baseline challenges of living with diabetes in Brazil are further exacerbated for socially vulnerable groups. Our findings highlight structural racism and poverty as critical social determinants of health. Adopting an intersectionality approach enables a more nuanced understanding of health inequities.

## Introduction

Diabetes mellitus is a major global public health challenge. In 2021, its prevalence among individuals aged 20–79 years reached 10.5% (536.6 million people) and is projected to increase to 12.2% (783.2 million) by 2045, with most affected individuals living in low- and middle-income countries [1]. In Brazil, the prevalence of self-reported diabetes increased from 6.2% in 2013 to 7.7% in 2019 among adults aged 18 years and over [2]. Furthermore, national estimates suggest substantial underdiagnosis and inadequate disease control, highlighting important gaps in diabetes care [3].

Diabetes and its complications are unevenly distributed across population groups. Women experience a greater burden of cardiovascular complications and mortality, whereas men are more frequently affected by microvascular complications [4,5]. Racial disparities are also evident, with black individuals presenting higher diabetes-related mortality and poorer clinical outcomes than white individuals [6,7]. Although socioeconomic disadvantage contributes to these inequalities [8], evidence suggests that structural racism plays an important role in shaping differential access to education, income, housing, and healthcare, thereby influencing diabetes risk and management [9,10].

Socioeconomic deprivation further affects diabetes self-management and access to healthcare. Financial constraints may limit access to medications, healthy diets, and healthcare services, contributing to poorer disease control and a higher risk of complications [11–13]. Although Brazil’s Unified Health System provides universal access to diabetes diagnosis and treatment, substantial inequalities remain in disease awareness, treatment, and glycemic control, particularly among socially vulnerable groups [14,15]. These disparities may contribute to worse health outcomes, including preventable complications and hospitalizations [16,17].

Intersectionality provides a useful framework for understanding how multiple social identities and systems of oppression interact to shape health outcomes [18,19]. By considering the combined effects of gender, race, and socioeconomic position, this approach can help identify population groups experiencing greater disadvantage in diabetes care.

Reflecting on the care provided to individuals with diabetes is essential for advancing health equity [20]. Therefore, this study aimed to evaluate the adequacy of care provided to individuals with diabetes in Brazil, considering the intersection of gender, race, and socioeconomic profile.

## Methods

### Data source and sample

This study used data from the 2019 PNS, conducted between 2019 and 2020 by the Brazilian Institute of Geography and Statistics (*Instituto Brasileiro de Geografia e* *Estatística* [IBGE]), in partnership with the Ministry of Health. The survey is a household-based, population-wide study designed to assess the determinants and conditionings of health, in addition to the healthcare needs of the Brazilian population, resulting in a nationally representative database.

PNS data are collected in person through household interviews and a sampling plan consisting of a master sample from the Integrated Household Survey System. This method ensures broad territorial coverage and adopts a three-stage cluster sampling design with simple random selection. The first stage comprises primary sampling units (census tracts), the second includes households within those tracts, and the third select residents aged 15 years or older from each household to complete the survey [21,22]. In 2019, a total of 108,457 households were selected, of which 100,541 were occupied, and 94,114 individuals consented to participate in the survey.

### Inclusion and exclusion criteria

For this analysis, participants aged 18 years or older who answered questions in module Q (chronic diseases) of the questionnaire were included. Respondents were included if reported a clinical diagnosis of diabetes based on an affirmative response to question Q30a (Has a doctor ever diagnosed you with diabetes?). Of the 94,114 individuals surveyed, 7,251 reported a medical diagnosis of diabetes. Of these, 148 women with gestational diabetes, 53 Indigenous individuals, 67 Asian descendants, and 15 individuals under 18 years of age were excluded. The final analytical sample comprised 6,967 individuals diagnosed with diabetes.

### Variables

#### Outcome variable.

The adequacy of care for individuals with diabetes in Brazil was assessed using 15 dichotomous items (No = 0; Yes = 1) regarding treatment and advice received during medical consultations, based on six questions of the PNS (Q46a, Q47a, Q48a, Q50, Q53, and Q54): (1- Q46a) Have you received guidance on maintaining a healthy diet?; (2- Q46a) Have you received guidance on maintaining an adequate body weight?; (3- Q46a) Have you received guidance to practice regular physical activity?; (4- Q46a) Have you been counseling to avoid smoking?; (5- Q46a) Have you been counseling to reduce excessive alcohol consumption?; (6- Q46a) Have you received guidance on reducing the consumption of pasta and bread?; (7- Q46a) Have you been advised to avoid sugar, sugary drinks, and sweets?; (8- Q46a) Have you received instructions on home blood glucose monitoring?; (9- Q46a) Have you been guided on regular foot examinations?; (10- Q47a) Request for a glycated hemoglobin (HbA1c) test during medical care?; (11- Q47a) Request a glycemic curve test?; (12- Q47a) Request cholesterol and/or triglyceride tests?; (13- Q50) Referral for consultation with a specialist physician in any of the consultations for diabetes?; (14- Q53) Had an eye or fundus exam in which your pupil was dilated in the last 24 months?; and (15- Q54) Has a doctor or health care provider undergone a foot examination for sores or lesions within the past 24 months?

Global scores were computed for each individual by summing the affirmative responses to the questions. Individuals with scores ≤12 points were classified as having low adequacy of care (i.e., did not receive a substantial portion of the essential clinical recommendations from national and international clinical guidelines for managing the disease) [23,24]. As literature lacks standardized international cutoff points for assessing adequacy of care for individuals with diabetes, the 75^th^ percentile was used as an empirical reference.

#### Exhibition variables.

The exposure variables were grouped as follows:

1. **Sociodemographic characteristics**: a) gender (man or woman); b) race (white or black/brown); c) age (18–44, 45–59, or 60 years or older); d) schooling (no schooling, complete/incomplete elementary school, complete/incomplete middle school, or complete/incomplete higher education); e) socioeconomic status, classified by household per capita income quintiles (1–5, from lowest to highest) and dichotomized into low (quintiles 1–3) and high socioeconomic status (quintiles 4 and 5); and f) area of residence (urban or rural). Additionally, an intercategorical approach based on the intersectionality theory [25] was used to create a variable combining gender, race, and socioeconomic profile.
2. **Health characteristics (yes or no answer):** a) history of any diabetes-related complication (heart attack, stroke, or other circulatory disorders, eye problems, kidney diseases, foot ulcer/wound, amputation of limbs [feet, legs, hands, or arms], diabetic coma, or other complication); b) previous hospitalization due to diabetes or some complication; c) diagnosis of another chronic disease (hypertension, dyslipidemia, heart disease, stroke, asthma, arthritis or rheumatism, chronic back pain, work-related illness, depression, other mental health condition, cancer, chronic lung disease, or chronic kidney disease); and d) self-rated health status (very good/good or fair/poor/very poor).
3. **Healthcare access characteristic (yes or no answer**)**:** possession of private health insurance.

### Statistical analysis

Given the complex sampling design of the survey, all analyses incorporated sample weight. The prevalence of each of the 15 adequacy of care indicators was calculated along with their corresponding 95% confidence intervals (95%CI). Subsequently, the prevalence of low adequacy of care was estimated according to each exposure variable, with a 95%CI. Bivariate Poisson regression was performed to estimate the crude prevalence ratio (PR) and corresponding 95%CI between low adequacy of care and sociodemographic, health, and access-related factors.

The multivariate Poisson regression model included exposure variables with p-value ≤ 0.200 in the bivariate analysis to estimate the adjusted PR. A hierarchical modeling strategy was employed, with variables entering the multivariate model in ascending order of their p-values. Only variables achieving statistical significance (p < 0.05) were retained in the final model. All analyses were conducted using Stata software version 13 (Stata Corp., College Station, United States).

No significant multicollinearity was detected among the independent variables included in the final multivariable model, as all Variance Inflation Factor (VIF) values were exceptionally low (ranging from 1.04 to 1.13).

The survey dataset is publicly available on the IBGE website. The 2019 PNS project was approved by the National Research Ethics Committee of the National Health Council, Ministry of Health, under Approval No. 3,529,376, dated August 23, 2019.

Given the complex sampling design of the survey, all analyses incorporated sample weight (Source: BGE. National Health Survey 2019: information on the sampling plan and weighting. Rio de Janeiro: Brazilian Institute of Geography and Statistics, 2020).

## Results

The sample of this study predominantly consisted of women (58.5%; 95%CI: 56.6 to 60.4) aged 60 years or older (60.8%; 95%CI: 59.0 to 62.7), who self-identified as black/brown (52.3%; 95%CI: 50.3 to 54.2), had completed elementary school (54.4%; 95%CI: 52.5 to 56.3), belonged to the income quintile 4 (23.3%; 95%CI: 21.6 to 25.1), and lived in urban areas (88.7%; 95%CI: 87.5 to 89.9). Most reported having at least one other chronic disease (85.6%; 95%CI: 84.3 to 86.8), and rated their health status as fair/poor/very poor (67.3%; 95%CI: 65.4 to 69.1). Despite this, 67.9% (95%CI: 66.1 to 69.7) had no complications related to diabetes, 87.2% (95%CI: 85.9 to 88.4) had not been hospitalized for the condition, and 72.1% (95%CI: 70.1 to 73.9) did not have private health insurance. The highest proportion of individuals with diabetes was observed among low-income black women (22.0%; 95%CI: 20.5 to 23.3), followed by high-income white men (12.6%; 95%CI: 11.2 to 14.1) and low-income black men (12.0%; 95%CI: 11.0 to 13.2) (Table 1).

**Table 1:** Distribution of individuals with a clinical diagnosis of diabetes according to sociodemographic characteristics, health conditions, and healthcare access. National Health Survey, Brazil, 2019 (n = 6,967).

Variable	N	% (95%CI)
**Gender**		
Men	2,828	41.5% (39.6 to 43.4)
Women	4,139	58.5% (56.6 to 60.4)
**Age**		
18 to 44 years	687	9.5% (8.4 to 10.6)
45 to 59 years	2,077	29.7% (28.0 to 31.5)
≤ 60 years	4,203	60.8% (59.0 to 62.7)
**Race/Skin Color**		
White	2,734	47.7% (45.8 to 49.7)
Black/Brown	4,233	52.3% (50.3 to 54.2)
**Schooling**		
No schooling	1,095	12.9% (11.8 to 14.2)
Elementary school	3,604	54.4% (52.5 to 56.3)
Middle school	1,455	21.0% (19.5 to 22.7)
Higher education	813	11.6% (10.5 to 12.9)
**Socioeconomic profile**		
Quintile 1	1,390	17.1% (15.8 to 18.5)
Quintile 2	1,193	16.3% (15.0 to 17.7)
Quintile 3	1,552	21.6% (20.1 to 23.2)
Quintile 4	1,433	23.3% (21.6 to 25.1)
Quintile 5	1,399	21.7% (20.0 to 23.4)
**Area of residence**		
Urban	5,631	88.7% (87.5 to 89.9)
Rural	1,336	11.3% (10.1 to 12.5)
**Complications due to diabetes**		
Yes	2,387	32.1% (30,.3 to 33.9)
No	4,580	67.9% (66.1 to 69.7)
**Hospitalization due to diabetes**		
Yes	933	12.8% (11.6 to 14.1)
No	6,034	87.2% (85.9 to 88.4)
**Diagnosis of another chronic disease**		
Yes	5,927	85.6% (84.3 to 86.8)
No	1,040	14.4% (13.2 to 15.7)
**Self-assessment of health**		
Very Good/Good	2,106	32.7% (30.9 to 34.6)
Fair/Poor/Very Poor	4,861	67.3% (65.4 to 69.1)
**Intercategories**		
Man, white, high-income	691	12.6% (11.2 to 14.1)
Man, white, low-income	513	8.7% (7.7 to 9.8)
Man, black, high-income	609	8.2% (7.3 to 9.2)
Man, black, low-income	1,015	12.0% (11.0 to 13.2)
Women, white, high-income	770	14.1% (12.8 to 15.6)
Women, white, low-income	760	12.3% (11.1 to 13.7)
Woman, black, high-income	762	10.1% (8.8 to 11.5)
Women, black, low-income	1,847	22.0% (20.5 to 23.4)
**Health insurance**		
No	5,275	72.1% (70.1 to 73.9)
Yes	1,692	27.9% (26.1 to 29.9)

The most frequently unmet aspects of diabetes care were related to foot care: 62.2% (95%CI: 60.3 to 64.1) had not undergone a foot examination by a healthcare professional in the previous 24 months, and 53% (95%CI: 51.0 to 54.9) had not received guidance to perform regular foot self-management. Additionally, 53.8% (95%CI: 51.7 to 55.8) reported not having been referred to a specialist, and 52.4% (95%CI: 50.3 to 4.5) had not been requested a glycemic curve test during medical consultations (Table 2).

**Table 2:** Prevalence of healthcare services not provided to individuals with diabetes in Brazil. National Health Survey, 2019.

Variables	Care not offered	
	N	Prevalence (95%CI)
Have you received guidance on maintaining a healthy diet?	1,096	16.5% (15.1 to18.0)
Have you received guidance on maintaining adequate body weight?	1,256	19.0% (17.5 to 20.6)
Have you received guidance to practice regular physical activity?	1,648	25.6% (24.0 to 27.4)
Have you been advised to avoid smoking?	2,470	37.0% (35.1 to 39.0)
Have you been advised to reduce excessive alcohol consumption?	2,485	37.1% (35.2 to 39.1)
Have you received guidance on reducing the consumption of pasta and bread?	1,463	22.2% (20.6 to 23.9)
Have you been advised to avoid sugar, sugary drinks, and sweets?	1,216	18.6% (17.1 to 20.2)
Have you received instructions on home blood glucose monitoring?	2,823	42.3% (40.4 to 44.2)
Have you been guided on doing regular foot examinations?	3,614	53.0% (51.0 to 54.9)
Request for a glycated hemoglobin (HbA1c) test during medical care?	2,922	39.8% (37.9 to 41.8)
Request a glycemic curve test?	3,748	52.4% (50.3 to 54.5)
Request cholesterol and/or triglyceride tests?	1,722	23.5% (22.0 to 25.2)
Referral for consultation with a specialist physician in any of the consultations for diabetes?	3,888	53.8% (51.7 to 55.8)
Have you had an eye or fundus exam in which your pupil was dilated in the last 24 months?	3,607	49.8% (47.8 to 51.8)
In the past 24 months, has a doctor or health care provider examined your feet for wounds or lesions?	4,436	62.2% (60.3 to 64.1)
Low adequacy of care for individuals with diabetes in Brazil (≤ 12 points)	5,052	72.2% (70.4 to 74.0)

In the bivariate analysis, the association between the low adequacy of care for individuals with diabetes and sociodemographic, health, and healthcare access characteristics revealed that all variables met the inclusion criterion (p < 0.20) and were included in the multivariate regression model. Notably, the intersection variable combining gender, race, and socioeconomic profile demonstrated a higher prevalence of low adequacy of care among black men and women with low income (79.6%; 95%CI: 75.7 to 83.1 and 79.1%; 95%CI: 76.2 to 81.8) (Table 3).

**Table 3:** Prevalence of low adequacy of care for individuals with diabetes in Brazil according to sociodemographic characteristics, health conditions, and healthcare access. National Health Survey, 2019 (n = 5,052).

Variables	Low adequacy of care for people with diabetes in Brazil		
	N	Prevalence (95%CI)	p-value
**Intercategories**			< 0.001
Men, white, high-income	418	62.9% (57.5 to 68.0)	
Men, white, low-income	389	77.1% (71.7 to 81.8)	
Men, black, high-income	407	66.5% (60.4 to 72.2)	
Men, black, low-income	814	79.6% (75.7 to 83.1)	
Women, white, high-income	511	67.0% (61.7 to 71.9)	
Women, white, low-income	567	75.1% (69.8 to 79.7)	
Women, black, high-income	490	64.5% (56.2 to 71.9)	
Women, black, low-income	1,456	79.1% (76.2 to 81.8)	
**Age**			0.177
18 to 44 years	530	77.1% (71.8 to 81.7)	
45 to 59 years	1,508	70.8% (66.9 to 74.5)	
≤ 60 years	3,014	72.2% (70.1 to 74.2)	
**Schooling**			< 0.001
No schooling	897	81.6% (77.7 to 85.0)	
Elementary school	2,738	75.1% (72.5 to 77.6)	
Middle school	940	66.6% (62.6 to 70.3)	
Higher education	477	58.6% (53.3 to 637)	
**Area of residence**			< 0.001
Urban	3,937	70.8% (68.7 to 72.7)	
Rural	1,115	83.9% (80.7 to 86.7)	
**Complications due to diabetes**			< 0.001
Yes	1,549	64.5% (60.9 to 67.9)	
No	3,503	75.9% (73.9 to 77.8)	
**Hospitalization due to diabetes**			< 0.001
Yes	584	62.7% (57.6 to 67.4)	
No	4,468	73.7% (71.7 to 75.5)	
**Diagnosis of another chronic disease**			0.055
Yes	4,286	71.6% (69.6 to 73.6)	
No	766	75.9% (71.9 to 79.5)	
**Self-assessment of health**			0.055
Very Good/Good	1,459	69.8% (66.6 to 72.7)	
Fair/Poor/Very Poor	3,593	73.5% (71.2 to 75.6)	
**Health insurance**			< 0.001
No	4,084	77.9% (75.7 to 79.9)	
Yes	968	57.8% (54.3 to 61.2)	

In the multivariate model, low adequacy of care remained associated with black men and woman with low income (PR = 1.10 and 1.09). The association was also found with the absence of diabetes-related complications (PR = 1.18) and no history of hospitalization due to diabetes (PR = 1.12). Factors associated with a lower prevalence of low adequacy of care included older age (45–59 years: PR = 0.90; ≥ 60 years: PR = 0.91), higher educational attainment (middle school: PR = 0.87; higher education: PR = 0.84), urban residence (PR = 0.92), and having private health insurance (PR = 0.78) (Table 4).

**Table 4:** Crude and adjusted prevalence ratio (PR) of low adequacy of care among individuals with diabetes in Brazil according to sociodemographic characteristics, health conditions, and healthcare access. National Health Survey, 2019 (n = 6,967).

Variables	Low adequacy of care for people with diabetes in Brazil			
	PR_crude_ (95%CI)	p-value	PR_adj_ (95%CI)	p-value
**Intercategories**				
Men, white, high-income	1.00		1.00	
Men, white, low-income	1.22 (1.10 to 1.36)	< 0.001	1.08 (0.98 to 1.20)	0.114
Men, black, high-income	1.05 (0.93 to 1.20)	0.379	1.00 (0.88 to 1.13)	0.960
Men, black, low-income	1.26 (1.15 to 1.39)	< 0.001	1.10 (1.00 to 1.21)	0.045
Women, white, high-income	1.06 (0.95 to 1.19)	0.273	1.05 (0.94 to 1.17)	0.349
Women, white, low-income	1.19 (1.07 to 1.32)	0.001	1.05 (0.95 to 1.17)	0.270
Woman, black, high-income	1.02 (0.88 to 1.18)	0.739	0.96 (0.83 to 1.10)	0.586
Women, black, low-income	1.25 (1.14 to 1.37)	< 0.001	1.09 (1.00 to 1.20)	0.039
**Age**				
18–44 years	1.00		1.00	
45–59 years	0.91 (0.84 to 0.99)	0.044	0.90 (0.84 to 0.97)	0.012
≤ 60 years	0.93 (0.87 to 1.00)	0.064	0.91 (0.84 to 0.97)	0.010
**Schooling**				
No schooling	1.00		1.00	
Elementary school	0.92 (0.87 to 0.97)	0.003	0.96 (0.91 to 1.01)	0.136
Middle school	0.81 (0.75 to 0.87)	< 0.001	0.87 (0.81 to 0.94)	0.001
Higher education	0.71 (0.64 to 0.79)	< 0.001	0.84 (0.76 to 0.94)	0.003
**Area of residence**				
Urban	0.84 (0.80 to 0.88)	< 0.001	0.92 (0.88 to 0.97)	0.001
Rural	1.00		1.00	
**Complications due to diabetes**				
Yes	1.00		1.00	
No	1.17 (1.10 to 1.24)	< 0.001	1.18 (1.11 to 1.25)	< 0.001
**Hospitalization due to diabetes**				
Yes	1.00		1.00	
No	1.17 (1.08 to 1.27)	< 0.001	1.12 (1.03 to 1.22)	0.004
**Diagnosis of another chronic disease**				
Yes	1.00			
No	1.05 (1.00 to 1.11)	0.044	–	
**Self-assessment of health**				
Very Good/Good	0.94 (0.90 to 1.00)	0.059	–	
Fair/Poor/Very Poor	1.00			
**Health insurance**				
No	1.00		1.00	
Yes	0.74 (0.69 to 0.79)	< 0.001	0.78 (0.73 to 0.84)	< 0.001

## Discussion

When evaluating the adequacy of care for people with diabetes in Brazil, based on the intersection of gender, race, and socioeconomic level, the findings of this study indicated that individuals who self-identified as black, regardless of gender, and low income with diabetes were more likely to receive low adequacy of care healthcare. Despite black individuals (sum of blacks and browns) representing the majority of the Brazilian population [19], disparities in healthcare adequacy remain persistent. In the United States, anti-black systemic racism has been identified in the literature as a primary determinant associated with health disparities between black and white individuals [26].

Our findings are consistent with previous studies suggesting that racism is an important determinant of inequities in diabetes care among marginalized and minority populations [27]. Furthermore, the intersection of racial and socioeconomic disadvantage may further amplify these inequities. Diabetes-related medical expenses are disproportionately concentrated in the low-income groups [28]. This group faces barriers to disease awareness, healthcare access, medication availability, and the effective management and prevention of diabetes complications [29].

According to Egede et al. [30], structural racism (the intersection of systemic, institutional, and structural factors) is broadly discussed in literature as influencing the health status of adults with diabetes. For example, structural racism has been significantly associated in previous studies with poorer clinical outcomes (HbA1c levels and blood pressure), suboptimal self-care behaviors, lower adherence to care standards, and higher mortality rates among this population.

Despite a recent increase in research on racism and health, the production of scientific knowledge in the health sciences remains insufficient [31,32]. The knowledge gaps regarding the health of black individuals risk undermining the implementation of the National Policy for the Integral Health of the Black Population, established by Ordinance 992/2009. This policy represents a milestone in acknowledging racism, ethnic-racial disparities, and structural racism as social determinants of health conditions, aiming to promote equity in healthcare and respond to the specificities of health-disease processes affecting black individuals in Brazil [33].

This study verified that the prevalence of diabetes, via an intercategorical approach based on the intersectionality perspective [25], was higher among women, individuals self-reported as black, and those with low income (22%; 95%CI: 20.5 to 23.4%). Similar results were found in a study conducted in Canada that also used an intersectional approach to assess health inequalities in individuals with diabetes: sex, racial identify, and income were associated with a greater risk of being diagnosed with diabetes [34].

In the bivariate and multivariate analyses, identifying as a man or woman, being black, and having low income were associated with lower adequacy of care for people with diabetes. A significant proportion of Brazilians report precarious access to healthcare services, particularly among the most socially vulnerable groups: black and brown individuals, rural residents, those who rate their health as poor/very poor, and those without private health insurance [35]. Palasson et al. [36] showed a similar pattern of lower healthcare adequacy for individuals with diabetes, where participants reported not receiving individualized treatment or preparation for self-management, alongside poor referral rates to specialists.

The most critical points related to the low adequacy of care for people with diabetes involved foot care, which is considered a fundamental strategy for preventing severe complications, including infections, ulcers, amputations, disabilities, and death. The 2023 International Working Group on the Diabetic Foot guideline recommends that individuals with diabetes with a very low risk of foot ulceration should do the annual screening, while those at higher risk screening should be screened every 1–3 months, coupled with structured education in foot care self-management [37]. However, according to data from the 2013 PNS, only 33.6% of Brazilians with diabetes had their feet examined by health professionals in the previous 12 months. The prevalence of never having undergone a foot examination was higher among the most vulnerable (rural residents, black individuals, and lower education levels) [38].

Despite government strategies promoting primary health care and chronic disease control policies, no meaningful improvements have been observed in the provision of foot care between the 2013 and 2019 PNS. Funding allocations and goals of government programs often prioritize other chronic diseases over diabetes. For example, diabetic foot complications, such as Charcot’s arthropathy and lower-limb amputation, have a five-year mortality rate higher than breast cancer and all cancers and also account for nearly one-third of the direct diabetes-related healthcare costs [39].

The present study also demonstrated that lower adequacy of care was more prevalent among individuals without complications or prior hospitalizations for diabetes. This finding reinforces the notion that primary healthcare delivery for chronic conditions in Brazil remains predominantly oriented toward curative care rather than prevention or health promotion, as observed in other studies [36,40]. In line with our findings, access to diabetes-related diagnostic tests in Brazil is higher among those aged 60 years and older [15,41], those with higher educational levels, urban residents, and individuals with private health insurance [15]. Older adults tend to have multiple chronic disease diagnoses requiring continuous care, which increases monitoring opportunities. In contrast, younger individuals typically perceive themselves as at lower health risk, are less symptomatic, and utilize healthcare services less frequently [15]. Individuals with private health insurance may also display better diabetes care outcomes because they access supplementary health services alongside the Unified Health System [42,43], a benefit that heavily extends to urban residents who have broader access to healthcare facilities and diagnostic resources [44].

Educational attainment also played a decisive role in the adequacy of diabetes care, as higher education levels were associated with improved self-management. Literacy and health literacy are essential for adherence to diabetes treatment [45]. According to Allen and McFarland [45], education provides social resources crucial for delaying disease onset, while income-based resources are more closely linked to disease management. Data from the ELSA-Brasil indicated that women with higher education presented mean values of ideal cardiovascular health (including the absence of diabetes) approximately 2.8 times higher than the expected value, with the proportion attributable to the combined effect of being a woman and having a high level of education reaching 63%. Moreover, black women and women with low education levels presented the worst results, indicating that overlapping social disadvantages are associated with greater cardiovascular vulnerability [46].

The literature demonstrates that intersectionality (i.e., interaction between social markers, such as race, gender, and socioeconomic status) yields different risk patterns for the development of chronic non-communicable diseases (NCDs) that are not captured by the analysis of isolated variables [47,48]. In a context where health equity cannot be understood without the concept of intersectionality [49], racism, sexism, and class inequalities constitute overlapping systems of oppression that structure the relative position of individuals in society and may be associated with variations in healthcare access, self-management practices, and chronic disease occurrences [46].

The Brazilian government presented the Strategic Action Plan to Combat NCD in Brazil (2011–2022) to the United Nations to achieve the Millennium Development Goals. This strategy established commitments and prioritized actions and investments to combat and manage chronic diseases, including diabetes and its risk factors [50]. Progress was observed and partial goals achieved, including increased physical activity, consumption of a healthy diet, and reduction of smoking. However, adult obesity increased, and the reduction rate in premature NCD-related mortality stagnated between 2015 and 2019 [44,51]. Therefore, the Strategic Action Plan to Combat NCD (2021–2030) was launched to intensify and ensure the continuity of the agenda to combat chronic diseases in Brazil. This plan includes the goals of reducing premature mortality due to NCD by one-third in the 30- to 69-year-old age group and the promotion of specific actions for diabetes prevention, surveillance, and comprehensive care [51]. Despite the lack of specific strategic actions for groups with greater vulnerability, the aim is to mitigate persistent disparities in healthcare access and adequacy for people with diabetes by strengthening policies that promote health equity for this population.

The limitations of this study include its cross-sectional design, which precludes establishing causal relationships or temporality between variables. Additionally, the self-reported nature of morbidity data is inherently dependent on healthcare access for diagnosis. Thus, limited access may have led to underestimating diabetes prevalence in the sample. Moreover, although the IBGE interviewers were trained to apply the survey, the self-reported nature of the required information and questions asking about behaviors from previous weeks may lead to memory bias. Differences in how interviewees interpret the questions are also possible. Furthermore, the “gender” variable in the IBGE questionnaire includes two categories (man and woman), disregarding gender identity. Gender is a spectrum and is particularly crucial for intersectionality analyses [52]. Likewise, indigenous and Asian descendants were excluded due to the small sample size, which precluded robust intersectional analyses involving these populations and limited the generalizability of our findings. Nonetheless, the importance of these groups in the debate on health inequalities is recognized, and specific studies that adequately address their realities are needed.

Moreover, the per capita household income variable was dichotomized into low (quintiles 1, 2, and 3) and high (quintiles 4 and 5) as it provides a concise representation of the Brazilian context, which is characterized by a higher concentration of the population in the lower-income strata [53]. The composite adequacy score assigned equal weight to each of the 15 indicators, although these components may differ in their clinical importance. As no validated weighting scheme currently exists for combining these indicators into a single measure of diabetes care adequacy, an equal-weight approach was adopted. This should be considered when interpreting the overall adequacy score. Finally, our composite measure of diabetes care adequacy assigned equal weight to each of the 15 indicators, although these components may differ in their relative clinical importance. As there is currently no validated weighting scheme for combining these indicators into a single measure of diabetes care adequacy, an unweighted approach was adopted to ensure transparency and reproducibility. Therefore, the overall adequacy score should be interpreted considering this methodological limitation.

The strength of this study is the focus on an intercategorical approach based on the intersectionality theory of social determinants of health to investigate the adequacy of care for individuals with diabetes. Our findings provide a national, population-based analysis that acknowledges the complex interactions between gender, race/skin color, and socioeconomic status, offering valuable insights to inform public policy.

## Conclusion

The baseline challenges of living with diabetes in Brazil appear to be greater for individuals belonging to socially vulnerable groups. Specifically, our findings highlight that vulnerabilities such as limited access to timely diagnosis and treatment, lower self-management preparedness, and structural barriers are more frequently reported among these populations. The observed health inequalities should be interpreted within the context of broader social determinants of health, including poverty and racial disparities.

Utilizing an intercategorical approach based on intersectionality theory allows for a clearer statistical understanding of health disparities and highlights the need for more inclusive, targeted intersectoral public policies to improve the Brazilian health system. Capacity building among health professionals focusing on the social dimensions of diabetes and its societal impacts may contribute to more effective and equitable interventions aimed at positively influencing the quality of life of this population.

## Supporting information

S1 Data Banco de dados (XLSX)
